# Trends in Transfusion Transmitted Infections Among Replacement Blood Donors in Karachi, Pakistan

**DOI:** 10.4274/Tjh.2012.0132

**Published:** 2013-06-05

**Authors:** Syed Mohammad Irfan, Jamal Uddin, Hasan Abbas Zaheer, Sadia Sultan, Amjad Baig

**Affiliations:** 1 Liaquat National Hospital, Department of Haematology, Karachi, Pakistan; 2 Pakistan Institute of Medical Sciences, Pakistan

**Keywords:** Replacement Blood Donors, Hepatitis-B, Hepatitis-C, HIV

## Abstract

**Objective:** To determine the prevalence of Hepatitis-B, Hepatitis-C and Human Immunodeficiency infections in replacement blood donors.

**Materials and Methods:** From January 2004 to December 2011, 108,598 apparently healthy donors donated blood at our Blood Bank. Screening was done by Microparticle Enzyme Immuno Assay (MEIA) method on Axsym System (Abbott Diagnostic, USA) and in year 2011 by Chemiluminescent Immunoassay (CIA) method on Architect i2000 (Abbott Diagnostic, USA). From 2010 onward, HIV reactive donors were advised for confirmatory tests and reported back with the results.

**Results:** Of the 108,598 total donors, 108,393 (99.8%) were replacement donors with a mean age of 28.92 (17-55) years. Of this, only 164 (0.15%) were females. Among the replacement donors, 4,906 (4.5%) were found to be reactive for Hepatitis-B, C and Human Immunodeficiency Virus. All the reactive patients, except one, were males. HbsAg was positive in 2,068 (1.90%) and anti-HCV in 2832 (2.61%) donors, while 111 (0.10%) were positive for Human Immunodeficiency Virus. Co-infectivity was observed in 103 (0.09%) cases. The prevalence appeared to be higher in younger age group (17-30 yrs). Only 16.6% cases should be patients returned with results of the confirmatory tests for HIV and were found positive.

**Conclusion:** Hepatitis-B and C sero-prevalence in our series of replacement donors appears high compared to most studies from neighboring countries and relatively low in comparison to earlier studies from Pakistan. Prevalence of HIV, however, appears low and turn out of HIV positive cases for confirmatory tests is low.

**Conflict of interest:**None declared.

## Abstract (tr)

**Amaç:** Kan bağışçılarında, Hepatit-B, Hepatit-C ve Human Immunodeficiency enfeksiyonlarının prevelansını belirlemek.

**Gereç ve Yöntemler:** Ocak 2004’den aralık 2011’e kadar, 108598 sağlıklı verici, kan bankamıza bağışta bulunmuştur. Taramalar, Axsym System (Abbott Diagnostic, USA) için Microparticle Enzyme Immuno Assay (MEIA) methodu ve 2011 yılında da Architect i2000 (Abbott Diagnostic, USA) için Chemiluminescent Immunoassay (CIA) yöntemi ile yapılmıştır. 2010 yılından itibaren, HIV reaktif vericilerin sonuçlarına doğrulama testleri uygulanmış ve kişilere geri bildirimde bulunulmuştur.

**Bulgular:** Toplam 108598 bağışçının 108393’ü (%99,8) replasman bağışçısı olup, yaş ortalaması 28,92 (17-55) idi. Bunların sadece 164’ü (%0,15) kadındı. Kan bağışçıları arasında 4906 (%4,5) olgu Hepatit-B, C ve Human Immunodeficiency Virus yönünden pozitif bulundu. Bunların biri hariç hepsi erkekti. Bağışçıların 2068’inde (%1,90) Hbs Ag ve 2832’sinde (%2,61) ise anti-HCV pozitif bulundu. 111 (%0,10) kişi Human Immunodeficiency Virus yönünden pozitifdi. Birden fazla enfeksiyon varlığı 103 (%0,09) olguda saptandı. Prevelans, görece genç yaştakilerde (17-30 yaş) daha yüksek bulundu. Sadece %16,6 olgu HIV doğrulama testi ile pozitif saptanarak reddedildi.

**Sonuç:** Çalışmamızda kan bağışçılarındaki Hepatit-B ve C seroprevelansı, komşu ülkelerin çalışmalarıyla kıyaslandığında yüksek bulunmakla birlikte, Pakistan’dan daha evvel yapılan çalışmalardaki sonuçlarla karşılaştırıldığında ise görece düşük saptanmıştır. Buna karşın, HIV prevelansı ve doğrulama testine tabi tutulan HIV pozitif olgu sayısı ise düşük görünmektedir.

## INTRODUCTION

Blood transfusion carries the risk of acquiring Transfusion Transmitted Infections (TTIs), including HIV, Hepatitis, Malaria, and infrequently Toxoplasmosis, Brucellosis and some other viral infections like CMV, EBV and Herpes. Among all infections, HIV and hepatitis are the most commonly screened viruses in a transfusion service [1].

Transfusion services in Pakistan are fragmented and there is great variation in the standard of services provided by the different blood banks in the country. There are some isolated centers which follow internationally recommended practices but on the whole there is a lot of room for improvement in the quality of work performed in the vast majority of the blood banks. Also we do not have a system to inform the health authorities about TTIs except for HIV positive cases, which are referred to provincial HIV / AIDS control program for confirmation and subsequent treatment if required. In the recent past, however, the level of awareness and sensitization about blood safety and particularly the significance of voluntary donations and screening of donated blood has improved considerably. Paid donations are becoming uncommon and the predominant reliance is on the replacement donors [2]. The prevalences of Hepatitis B, C and HIV show wide variation in the general public and blood donors [3,4,5]. 

In several studies conducted in Pakistan and in other countries of the world, prevalence of TTIs appears high among replacement blood donors compared to voluntary donors [6,7]. Prevalence of TTIs is highly uncommon in the developed countries due to a well-developed healthcare and blood transfusion system which follows stringent donor selection criteria, deferral on the basis of high risk behavior and sensitive screening tests [8]. 

We undertook a review of sero-prevalence of blood donors in our hospital. Being the largest study on replacement blood donors from Pakistan, it is likely to reflect trends in TTIs in major cities of the country. 

## MATERIALS AND METHODS

This cross sectional study was conducted in the blood bank of Liaquat National Hospital from January 2004 to December 2011. Institutional clearance was obtained from the ethical committee of the Hospital. 

Donors were interviewed and an informed consent was sought from all of them. The findings were recorded with a on a specially designed questionnaire which was further improved from 2010 onwards. Donor’s name, age, sex, national identity card number, phone number and type of donor (replacement or voluntary) were recorded. Only healthy blood donors, having no history of jaundice, intravenous drug abuse, non-marital sexual contacts, tattooing, blood transfusion and fulfilling the physical fitness criteria for age, weight, or blood pressure and haemoglobin levels were bled for donation. Screening methods included Microparticle Enzyme Immunoassay on Axsym system (Abbott diagnostic, USA) and in year 2011 by Chemiluminescent Immunoassay (CIA) method on Architect i2000 (Abbott Diagnostic, USA). Reactive results were repeated on the same sample with the same method. From 2010 onward, data were collected prospectively and HIV positive patients were contacted on cell phone, counseled and referred to provincial HIV/AIDS control centers for confirmation and subsequent therapy if required. Donors were advised to come back to us with results of confirmatory tests. Donors were contacted multiple times if they did not respond initially. 

Data were entered and analyzed using SPSS version 13 statistical package. Mean ± SD was calculated for the quantitative variable, i.e. age. Frequency and percentages were calculated for qualitative variables, i.e. sex, type of donor and positivity rate. 

## RESULTS

**Figure 1:**
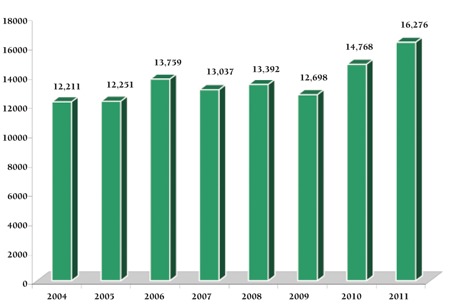
Replacement donors over years.

A total of 108,598 blood donations were collected during the study period. Blood donation rate remained similar over years with an increasing trend from 2010 onward. Of the total donors, 108,393 (98.8 %) were replacement donors and only 205 (1.2%) were voluntary donors (Figure 1). Mean age of replacement donors was 28.92 years and all except 164 (0.15%) were males. 

**Figure 2:**
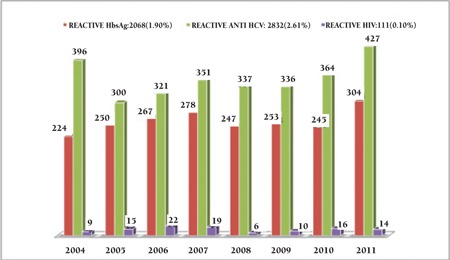
Distribution of reactive donors per year.

**Figure 3:**
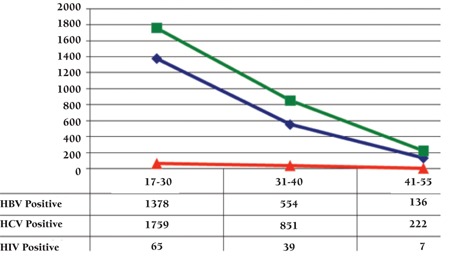
Age groups and seroprevalence of Hepatitis-B, C and HIV.

The positivity rates among replacement and voluntary donors were 4.51% and 0.48%, respectively. Among the replacement donors sero-prevalence of Hepatitis B, C and HIV was 2068 (1.90%), 2832 (2.61%) and 111 (0.10%) cases, respectively. Positivity rate for viral markers remained the same over study years except for HIV which showed a small dip in the year 2008 (Figure 2). Little difference was seen in the mean age of Hepatitis B (28.5 years), Hepatitis C (29.4 years) and HIV positive cases (30.0 years). Hepatitis B, Hepatitis C and HIV reactivity rates were found high among the younger age group (Figure 3). Co-infectivity was seen in 103 donors (0.09%). Co-infectivity for Hepatitis B and Hepatitis C was highest: 0.084%. HIV seropositivity along with hepatitis B and C was present in 0.004% and 0.001%, respectively, and reactivity for all three viral markers was seen in 0.001% donors (Table 1). 

Of the 30 HIV reactive patients from 2010 onward, 28 could be contacted on cell phone for counseling and confirmatory tests while 10/30 donors (33%) did not respond to calls at all. Only 60% received the calls, however, and only 12/30 (40%) reported to blood bank for detailed history and were referred to designated centers for confirmatory tests. They were also advised to report back to us with the results of confirmatory tests to keep our record updated. However, only 5/30 (16.6%) returned back and all were positive for the confirmatory tests for HIV. 

## DISCUSSION

Liaquat National Hospital is a 700 bed tertiary care hospital, located in the heart of cosmopolitan city of Karachi (population 18 million). The hospital caters to a population of about 4 million people. Majority of the patients (and donors) attending the hospital come from lower middle class compared to public care hospitals, which caters to patients from a low socioeconomic group.

Blood donation in Pakistan, like most of the developing world, is predominantly reliant on family / replacement donations. This category is known to have higher prevalence of TTIs compared to regular voluntary donors [6,7]. However, for cultural, economic and social reasons replacement donors cannot and should not be discouraged or eliminated from the blood donor pool especially in countries like Pakistan which has a strong tradition of family bonds. The replacement donors in such societies should be motivated and recruited to become regular voluntary donors to ameliorate the chronic shortage of voluntary blood donors. Thus, in such an environment the strategy for mobilization, recruitment and retention of voluntary non-remunerated blood donors should focus on the captive audience, i.e. the replacement blood donors [9]. This approach is likely to be more beneficial as well as more cost-effective than a generalized media campaign for the general public, adopted conventionally in the developed countries which do not have a large pool of replacement donors available. 

Hepatitis B and C remain major public health concern in Pakistan. In our study prevalence of Hepatitis B and Hepatitis C was relatively on the lower side compared to many studies from Pakistan [3,4,6,7]. Mujeeb and Mehmood reported high positivity rate of hepatitis B (4.9%) and comparable rate for hepatitis C (2.4%) from Karachi in family / replacement donor [6]. Our results for hepatitis B and C are also low when compared to another study in replacement donors by Sultan et al. from Lahore, the 2nd largest city of Pakistan [7]. Mujeeb et al [10] has reported higher prevalence of hepatitis B (4.7%) and C (3.6 %) in first time replacement donors from the same city. Low prevalence in our series of donors is likely because of stringent behavioral screening practices which we have been trying in last few years. We specifically paid more attention to improved donor selection criteria, education and salary package of donor attendants and supervision of the donor area by trained resident doctors. Comparative data in replacement donors from neighboring countries are shown in Table 2 which exhibits higher prevalence of hepatitis B and C in our series of donors [11,12,13,14,15]. Likely reasons are deepened poverty, statutory ambiguity in certain laws, unsafe health practices of public & professionals and lack of universal access to health care. Also our prevalence for hepatitis B and C appear high in comparison to studies on voluntary donors from the neighboring countries which is self explanatory [12,13,16] (Table 3). Wide variations are seen in positivity rate for TTIs from other developing countries of the world for which varied and multiple reasons exist. Our positivity rates are much higher when compared to developed nations where greater awareness, high living standards and use of nucleic acid amplification testing (NAT) have led to very low risk of acquiring TTIs [17,18,19,20].

HIV positivity rate in our study, however, appear significantly low when compared to studies from neighboring countries in replacement donors except for a study by Ahmed et al., which reported a low HIV positivity rate of 0.008% [11,13,14,15]. Also the trends appear static over years (except for a dip in 2008). Another striking feature in our study is the high prevalence of TTIs in youngest age group (Figure 3). Sultan et al., which reported a significantly lower prevalence of HIV in replacement donors younger than 35 years (0.03%) versus 35–55 years (0.3%) [7] . The same study has reported lower prevalence of hepatitis B and hepatitis C in age group younger than 35 years (1.4% and 3.3%) compared to donors with 35-55 years of age (2.6% and 6.3%). The higher prevalence of TTIs in younger donors as seen in our study can emerge as an immense public health problem as well as it may lead to higher donor deferral rates and so shortage of blood in future. However the issue has been actively taken up by provincial government and a “hepatitis prevention and control program” has been launched aggressively in last 2-3 years. Public awareness is being raised and poor people are being provided Hepatitis vaccine free of cost. As a result of extensive campaigns, people are getting vaccinated privately for their health and safety. However the trend needs to be investigated by public health experts as there may be a need to invest in promotion of healthy life style. 

Multiple infections pose a small but enhanced risk to the recipient’s life. Co-infectivity rates in blood donors have not been reported from Pakistan and limited data are available from the neighboring countries. In our series of donors, co-infectivity was detected in 103 (0.09%) donors. Hepatitis B & C co-infection was seen in 94 (92.2%) compared to HIV and Hepatitis co-infection which was seen in 8 (7.8%) donors only (Table 1). Kaur et al. from India also show low rates for co-infection in HIV seropositive donors compared to those who were seronegative for HIV [13]. Studies from Sub-Saharan Africa also show high positivity rate of HIV co-infections in countries which are likely to have a Generalized HIV Epidemic [9]. A trend towards higher co-infectivity also prevails in replacement donors compared to voluntary donors [13].

As a conclusion it appears that Hepatitis B and C reactivity rates in our series of donors appear high compared to most reported studies in replacement donors from neighboring countries, however, HIV appears low. Higher prevalence in younger donors is alarming and is likely to adversely affect the current static trends in medium and long term. This situation calls for concerted efforts to overcome it as it implies that we are likely to face more shortage of healthy donors in future. 

## ACKNOWLEDGEMENTS

The authors sincerely thank blood donors for giving correct information. We are also thankful to Mr. Mustansar for data analysis and Mr. Asif for Manuscript typing. 

## CONFLICT OF INTEREST STATEMENT

The authors of this paper have no conflicts of interest, including specific financial interests, relationships, and/ or affiliations relevant to the subject matter or materials included. 

## Figures and Tables

**Table 1:**
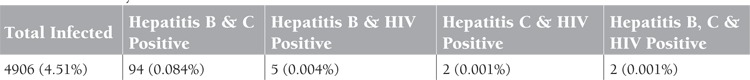
Co-infectivity of the TTIs.

**Table 2:**
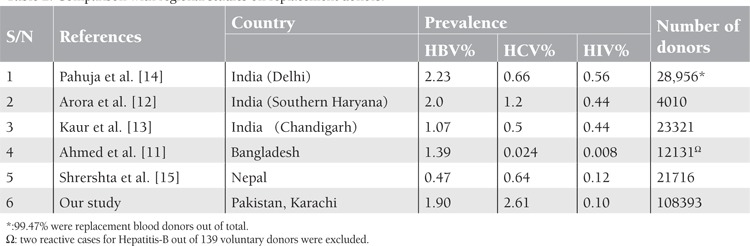
Comparison with regional studies on replacement donors.

**Table 3:**
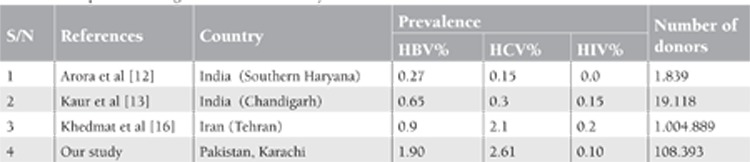
Comparison with regional studies in voluntary donors.
